# Diagnostic Performance of PD-L1 versus PD-1 Expression in Circulating CD20 Cells in Diffuse Large B-Cell Lymphoma

**DOI:** 10.3390/antib11010015

**Published:** 2022-02-16

**Authors:** Manal Mohamed Saber

**Affiliations:** Clinical Pathology Department, Faculty of Medicine, Minia University, Minia 61519, Egypt; manal.saber@mu.edu.eg; Tel.: +20-106-090-6673

**Keywords:** PD-L1, PD-1, CD20, DLBCL

## Abstract

This study aimed to investigate PD-L1 and PD-1 expression in circulating CD20+ cells in diffuse larger B-cell lymphoma (DLBCL) and to evaluate the predictive and diagnostic performance of PD-L1 versus PD-1 expression in circulating CD20+ cells in DLBCL. Percentages of CD20+, PD-L1+CD20+, and PD-1+CD20+ cells were measured by flow cytometry in 40 DLBCL blood samples and 19 healthy controls. The DLBCL patient group was subdivided into 20 newly diagnosed patients with no treatment yet and 20 patients that had finished six cycles of CHOP therapy. Percentages of PD-L1+CD20+ and PD-1+CD20+ cells were highly significantly increased in pre-therapy patients in comparison to healthy volunteers (*p* < 0.001). Meanwhile, a significant decrease in percentages of PD-L1+CD20+ and PD-1+CD20+ was observed in post-CHOP therapy patients in comparison to pre-therapy patients (*p* < 0.001). PD-L1+CD20+ cells were significantly decreased in post-therapy patients when compared to normal controls (*p* < 0.001), while not for PD-1+CD20+ cells. A strong significant positive correlation between percentages of PD-L1+CD20+ and PD-1+CD20+ was detected in DLBCL patients (*p* < 0.001). In the pre-therapy group, high PD-L1+CD20+ and PD-1+CD20+ percentages were correlated with serum LDH levels (*p* = 0.021, *p* < 0.001). High percentages of PD-1+CD20+ were found in DLBCL patients with splenomegaly (*p* = 0.027). The results revealed that patients with advanced tumor stages, poor ECOG performance, and non-GCB DLBCL type had increased percentages of PD-L1+CD20+ and PD-1+CD20+ cells. Moreover, PD-L1+CD20+ % and PD-1+CD20+ % were significantly increased in DLBCL patients with bone marrow involvement or B symptoms. The superiority of PD-L1+CD20+ over PD-1+CD20+ was more profound in DLBCL prediction [AUC: 1.0] and in discriminating newly diagnosed patients [AUC: 1.0]. The findings suggest that increased PD-L1/PD-1 expression in peripheral CD20 cells may serve as a companion diagnostic marker for DLBCL. Moreover, percentages of PD-L1+CD20+ cells have better diagnostic performance with higher sensitivity and specificity than PD-1+CD20+ %.

## 1. Introduction

Diffuse large B-cell lymphoma (DLBCL) is the prevalent type of non-Hodgkin lymphoma (NHL), representing about 37% of NHL patients [1,2,3]. DLBCL is an aggressive type of lymphoma accompanied by significant heterogeneity in molecular genetics and clinicopathological features [4]. DLBCL is categorized into two types: germinal center B-cell (GCB type) and non-GCB type [1,5,6]. The standard protocol treatment for DLBCL is cyclophosphamide, vincristine, doxorubicin, and prednisone, named “CHOP” [7]. Patients who received CHOP therapy had 55.8% 6 years survival, and those who had R-CHOP had a 74.3% survival rate [8]. Extensive research to try to help improve outcomes for these patients is ongoing [9].

The CD20 antigen is a 33–37-kDa non-glycosylated phosphoprotein present on the mature B-cell surfaces [10]. CD20 expression begins at the pre-B-cell and continues until its terminal differentiation into plasma cells [11]. CD20 has been assumed to have a role in B-lymphocyte growth, differentiation, signaling of B-cell receptor (BCR), and the initiation events of the cell cycle [12,13]. Many studies showed a high level of CD20 in lymphomas, Hodgkin lymphoma (HD), and DLBCL [14,15,16]. Almost all DLBCL are positive for CD20 [17]. CD20-negative DLBCL is rare [18] and has been reported to have worse outcomes compared to other DLBCL [19,20,21]. Several reports have demonstrated that CD20-positive DLBCL attained a complete remission rate of about 60% when treated with CHOP alone [22,23,24,25]. CD20 expression in DLBCL cells and HRS cells acted as a guide for the treatment of Hodgkin lymphoma (HD) and DLBCL patients with regimens including chemotherapy and anti-CD20 antibody [17,26]. The addition of rituximab (monoclonal antibody against CD20), to cyclophosphamide, doxorubicin, vincristine, and prednisone (CHOP) has improved DLBCL patients’ survival [27,28], however, 30–40% of cases still develop into relapsed/refractory (R/R) disease, suggesting a selective process toward increased resistance [29,30].

Programmed cell death ligand 1 (PD-L1), a 40-kDa inhibitory protein, was known to be engaged in inhibiting immune responses [31,32]. PD-L1 binding to programmed cell death 1 (PD-1) can result in immune evasion of tumors [33]. PD-L1 and PD-1 were found to be present on B lymphocytes [34,35,36]. PD-1/PD-L1 expression has been reported in DLBCL patients [37,38,39,40,41]. PD-L1/PD-1 blockage elucidated advantageous therapy in B-lymphoma patients [42,43]. Previously, it was reported that the immune responses might be regulated through the PD-1/PD-L1 mechanism. This paper investigated PD-1/PD-L1 expression in circulating CD20+ cells and found some associations with laboratory and clinicopathological parameters in DLBCL. The study also evaluated the possibility of PD-L1+CD20 and PD-1+CD20+ cells as references for early diagnosis of DLBCL.

## 2. Materials and Methods

### 2.1. Patients

This study included 19 healthy volunteers and 40 patients diagnosed with DLBCL. Patients were selected from Minia Oncology Center. DLBCL diagnosis was confirmed by pathological examinations, immunophenotyping, and radiological analyses. Patient groups were categorized into 20 newly diagnosed DLBCL patients and 20 patients who had received 6 cycles of CHOP (cyclophosphamide, doxorubicin, oncovin, prednisone) treatment [44]. DLBCL diagnosis was confirmed by immunophenotyping and histopathology. Healthy controls were matched for age and sex. Written consent was obtained from every subject. 

### 2.2. Clinical Criteria and Laboratory Samples Collection

Lymph node biopsy, bone marrow aspiration, and full clinical examination were performed to define the type, stage, and clinical evaluation of DLBCL. According to WHO classifications, three experienced pathologists validated DLBCL pathological specimens [45]. DLBCL classification into GCB and non-GCB types were performed by using the histomorphological data [45], followed by the application of Han’s Algorithm [46]. Immunophenotyping of DLBCL was assessed using a flow cytometer. Pelviabdominal ultrasound and X-ray were performed for DLBCL patients to identify extramedullary involvement. DLBCL evaluation was performed regarding the Eastern Cooperative Oncology Group scale [47]. DLBCL staging was performed using the Ann Arbor system through positron emission tomography scans or computed tomography [48]. The patients who had incomplete clinical or pathological data were not involved in the study. Healthy volunteers who had chronic infections or autoimmune diseases were excluded. On a sterile K3EDTA tube, 2 mL of blood were used for the determination of flow cytometric analysis and complete blood count (CBC). Furthermore, on a plain tube, 4 mL of blood were centrifuged, and the expressed serum was used for the determination of blood glucose levels, LDH, blood urea, and liver functions tests (total bilirubin, ALT, AST, and serum albumin).

### 2.3. Antibodies

PD-L1, PD-1, and CD20 expression was determined using Flow Cytometry analysis with commercially available, validated PD-L1 antibody: 29E.2A3, monoclonal antibody (BioLegend; San Diego, CA, USA, catalog No. 309706) [49,50], PD-1 antibody, EH12.2H7, monoclonal antibody (BioLegend; catalog No. 329906) [51,52], and CD20, 2H7, monoclonal antibody (BioLegend; catalog No. 302304) [53,54]. Each antibody was validated for flow cytometric analysis by proving expression by blocking, immunohistochemistry (IHC), and immunoprecipitation [49,50,51,52,53,54].

### 2.4. Flow Cytometry Analysis

Identification of PD-L1+CD20+ % and PD-1+CD20+ % was assessed. Incubation of peripheral blood sample was performed with isotype-matched control or monoclonal antibodies. For each sample, 3 tubes were labeled, one tube for phycoerythrin (PE) conjugated anti-CD 274, fluorescein isothiocyanate (FITC)-conjugated anti-CD20, one tube for PE-conjugated anti-CD 279, FITC-conjugated anti-CD20, and the other tube was used for negative isotypic control. All antibodies were purchased from BioLegend (San Diego, CA, USA). Briefly, 5 μL of antibodies were added to 100 μL of peripheral blood and were incubated for 20 minutes in the dark at room temperature. Then, 2 mL red cell lysis buffer was added, vortexed, and incubated for 15 minutes in the dark at room temperature. Sample centrifugation was performed for about five minutes at 1200 rpm, and the supernatant was removed. Subsequently, 1 mL of washing phosphate-buffered saline (PBS) solution was added to every tube, mixed, and centrifuged at 1200 rpm for five minutes and the supernatant was removed. Then, 300 μL PBS was added to the cells to resuspend them for flow cytometry analysis. BD-FACS FLOW (Argon laser, BD Biosciences, San Jose, CA, USA) was used for cells analysis using the Cell Quest Program. The lymphocyte gating was assessed according to the forward scatter vs. side scatter (FSC/SSC) plot. CD20+ %, PD-L1+CD20+ %, and PD-1+CD20+ % cells were assessed. 

### 2.5. Laboratory Methods

CBC was analyzed by using a Sysmex KX-21N (TAO Medical incorporation, Chuo-ku, Kobe, Japan) automated cell analyzer. Blood urea, creatinine, alanine aminotransferase (ALT), total bilirubin, LDH, blood glucose levels, albumin, and aspartate aminotransferase (AST) were analyzed by using the auto-analyzer clinical chemistry system (Schiaparelli Biosystem INC, Fairfield City, NJ, USA).

### 2.6. Statistics

Data analyses were undertaken using the SPSS program (SPSS–25, Chicago, IL, USA). Data were expressed by the interquartile range (IQR) and median. Mann–Whitney test was performed for data analysis of non-parametric quantitative data between two groups. For non-parametric quantitative results among more than two groups, the Kruskal Wallis analysis was used followed by pairwise comparisons between every two groups using Bonferroni correction. The Chi-square analysis was used for qualitative data between the two groups (if up to 20% of cells have an expected count less than 5). The Fisher exact test was performed for qualitative data between the two groups (if >20% of cells have expected count less than 5), and for qualitative data between more than two groups and between every two groups. The ROC curve was assessed for data analysis. The association between two continuous variables was determined using Pearson’s correlation coefficient, while Spearman’s correlation coefficient was determined for the association between qualitative ordinal and continuous variables. Significant differences are identified by an asterisk (*) (*p* < 0.05). Highly significant differences are identified by an asterisk (**) (*p* < 0.001).

## 3. Results

### 3.1. Patients’ Characteristics

Characteristics of 40 DLBCL patients (20 male, 20 female) and 19 healthy volunteers are illustrated in Table 1. DLBCL patients had a median age of 46.5 years (the range: 34–55.8). The age and gender of the patients were matched with that of the healthy controls. No significant difference was observed between controls and DLBCL patients regarding their gender and ages (*p* > 0.05). A highly statistically significant difference between patients and controls regarding hepatomegaly and splenomegaly was observed (*p* < 0.001). Of all 40 DLBCL patients, 20 newly diagnosed DLBCL patients and 20 patients had finished CHOP therapy.

### 3.2. PD-L1/PD-1 Expression in Peripheral CD20+ in DLBCL

To measure PD-L1+CD20+ % and PD-1+CD20+ %, flow cytometry was employed. Increased PD-L1+CD20+ % were observed in DLBCL patients compared to healthy volunteers (median 9.5 [range: 6.7–16] % vs. median 1.0 [range: 0.8–1] %, *p* < 0.001). As well, PD-1+CD20+ % of cells was also significantly increased in patients in comparison to normal volunteers (median 4.1 [range: 0.9–9] % vs. median 0.7 [range: 0.6–1] %, *p* = 0.001) as shown in Table 2.

**Figure 1:**
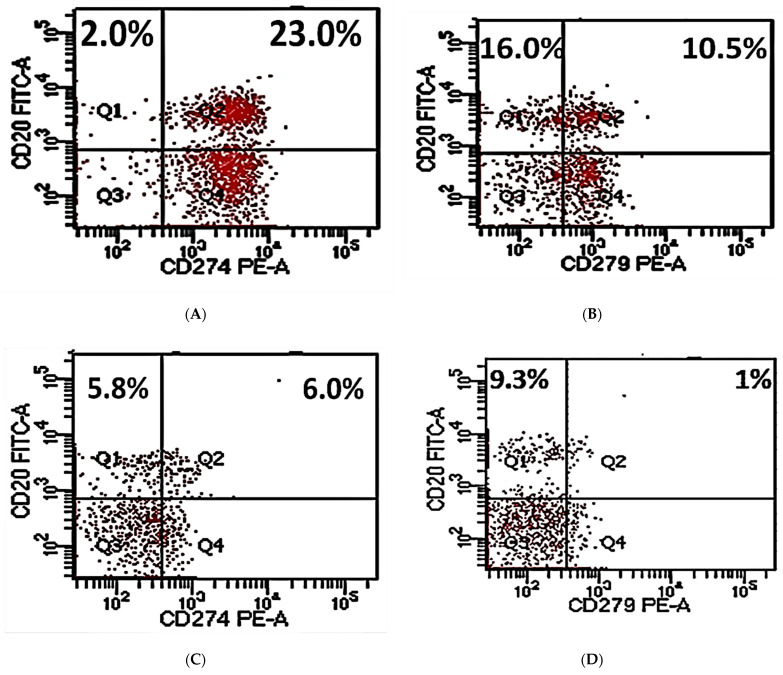

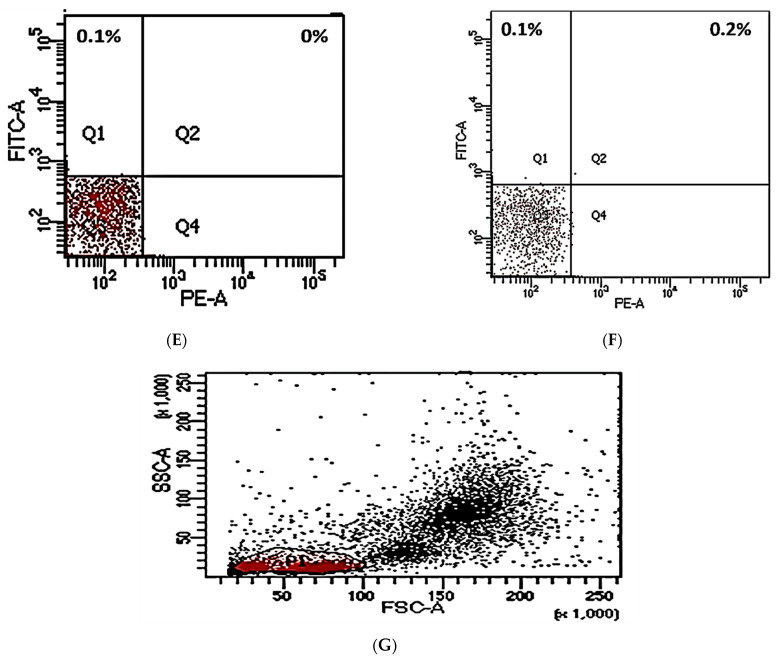
Flow cytometric plots for measurements of CD20+ %, PD-L1+CD20+ %, and PD-1+CD20+ % of cells. CD20+ %, PD-L1+CD20+ %, and PD-1+CD20+ % within pre-therapy DLBCL patients (**A**,**B**); CD20+ %, PD-L1+CD20+ %, and PD-1+CD20+ % within post-therapy DLBCL patients (**C**,**D**); isotype-matched control for CD20 and PD-L1 (**E**); isotype-matched control for CD20 and PD-1 (**F**); gating used to detect cells for analysis (**G**).

PD-L1+CD20+ % and PD-1+CD20+ % were then analyzed in 20 pre-therapy patients and 20 post-therapy patients **(**Figure 1). Newly diagnosed patients had CD20+ percentages ranging from 18.3 to 26.8 with a median of 22. The range of PD-L1+CD20+ % is 16–25 with a median of 16 while the percentages of PD-1+CD20+ ranged from 8 to 11 with a median of 9 (Figure 1, Table 3). PD-L1+CD20+ % and PD-1+CD20+ % cells were highly significant in DLBCL patients compared to normal controls (*p* < 0.001). Newly diagnosed patients and controls did not exhibit any significant difference regarding CD20 % of cells (*p* > 0.05).

Post-therapy patients had percentages of CD20+ cells ranging from 9.6 to 11.8 with a median of 10. They also had percentages of PD-L1+CD20+ cells ranging from 6 to 7.9 with a median of 6.8. PD-1+CD20+ % ranged from 0.6 to 1.0 with a median of 0.7 (*p* < 0.001) (Figure 1, Table 3). Percentages of CD20+ and PD-L1+CD20+ cells were decreased significantly in post-therapy patients in comparison to pre-therapy patients and normal volunteers (*p* < 0.001). PD-1+CD20+ % was significantly decreased in post-therapy DLBCL patients compared to newly diagnosed ones (*p* < 0.001). Interestingly, no significant difference was detected between post-therapy patients and normal controls regarding PD-1+CD20+ percentages (*p* > 0.05) (Table 3).

In 59 subjects, we found percentages of CD20+ cells >19 in 15 healthy controls (78.9%) in group I, 13 subjects (65%) in group II, and 0 subjects (0%) in group III. Percentages of PD-L1+CD20+ cells were >6.8 in 0 controls (0%) in group I, 20 subjects (100%) in group II, and 10 subjects (50%) in group III. PD-1+CD20+ % of cells was >1 in 2 healthy volunteers (10.5%) in group I, 20 subjects (100%) in group II, and 2 subjects (10%) in group III (Table 4).

### 3.3. PD-L1/PD-1 Expression and Laboratory-Systemic Data

**Figure 2:**
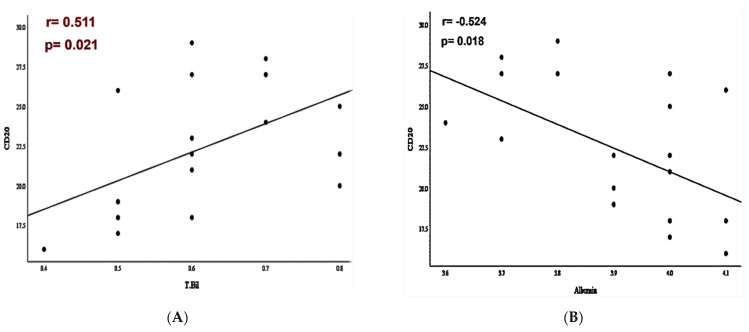

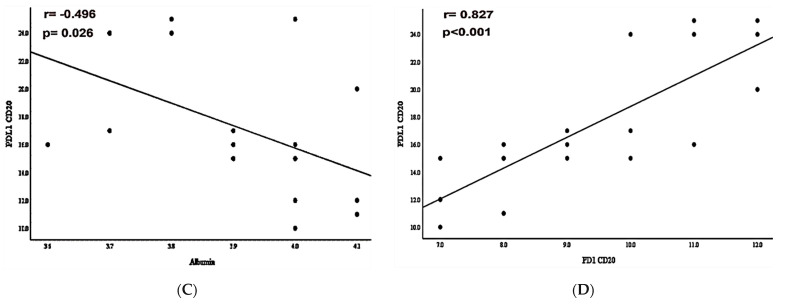
(**A**) Diagram illustrating the positive association between percentages of CD20+ cells and total bilirubin levels (r = 0.511; *p* = 0.021); (**B**) diagram illustrating the negative association between percentages of CD20+ cells and serum albumin levels (r = −0.524; *p* = 0.018); (**C**) diagram illustrating the negative association between percentages of PD-L1+CD20+ cells and serum albumin levels (r = −0.496; *p* < 0.026); (**D**) diagram illustrating the positive correlation between PD-L1+CD20+ % and PD-1+CD20+ % (r = 0.827; *p* < 0.001). Pearson’s correlation analysis was used.

In newly diagnosed patients, a significant positive association between CD20+ percentages and total bilirubin was found (r = 0.611 and *p* = 0.021) (Figure 2A). Additionally, CD20+ % and PD-L1+CD20+ % of cells had a highly significant negative correlation with albumin (*p* = 0.018 and *p* = 0.026) (Figure 2B,C). The association between PD-L1+CD20+ % and PD-1+CD20+ % was further investigated in DLBCL patients (Figure 2D). The analysis identified that PD-L1+CD20+ % cells were positively associated with the percentages of PD-1+CD20+ cells (*p* < 0.001). No significant associations were detected in post-therapy patients (splenomegaly, hepatomegaly, or laboratory profile).

A strong significant association was found between high CD20+ %, PD-L1+CD20+ %, PD-1+CD20+ %, and serum LDH levels (*p* = 0.002, *p* = 0.021; *p* < 0.001). Additionally, a significant positive association was identified between high PD-L1+CD20 percentages and random blood sugar (*p* = 0.020). Interestingly, those with splenomegaly had high significant percentages of PD-1+CD20+ cells (*p* = 0.027) (Table 5).

### 3.4. Clinicopathological Analysis and PD-L1/PD-1 Expressions in DLBCL

**Figure 3:**
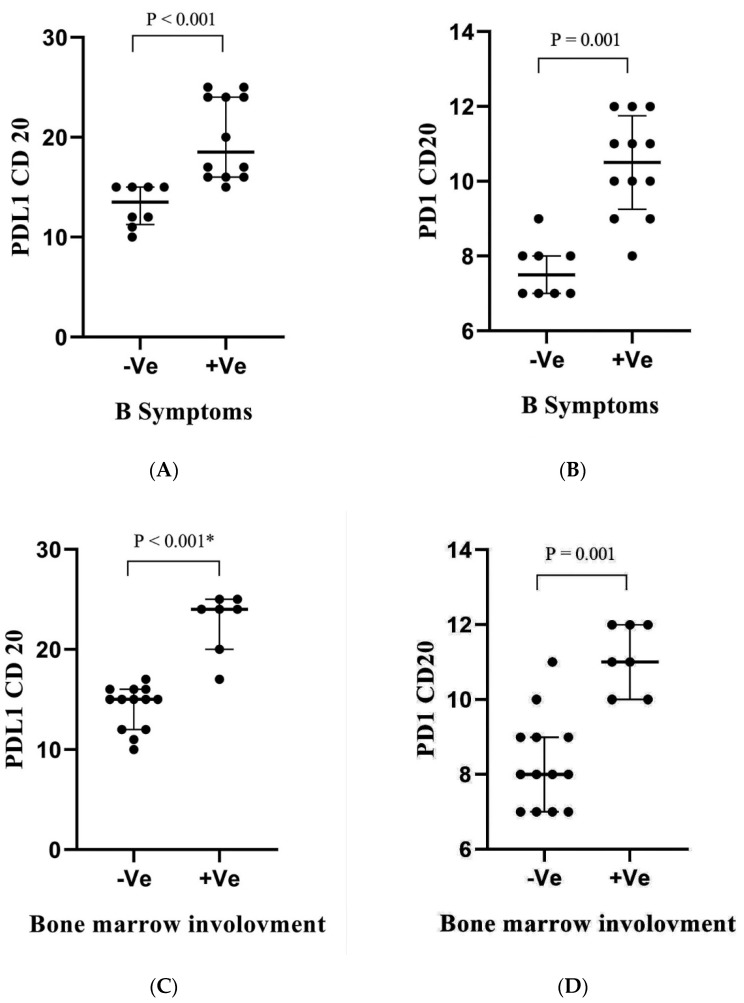

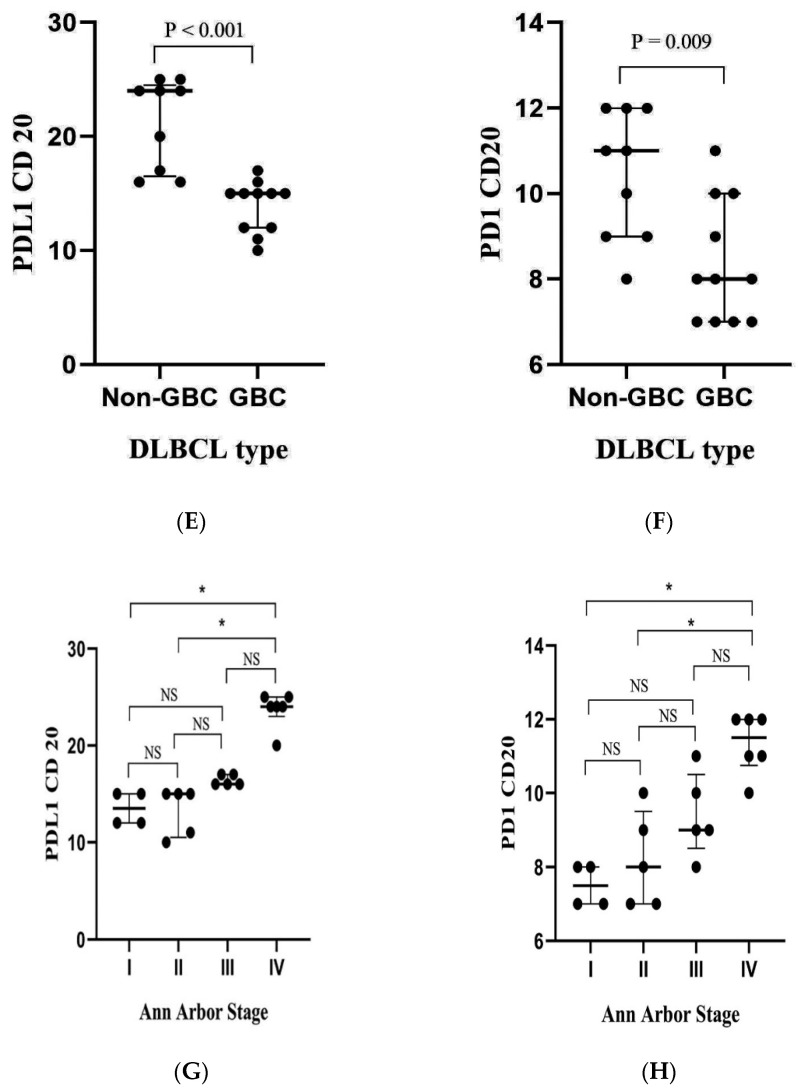

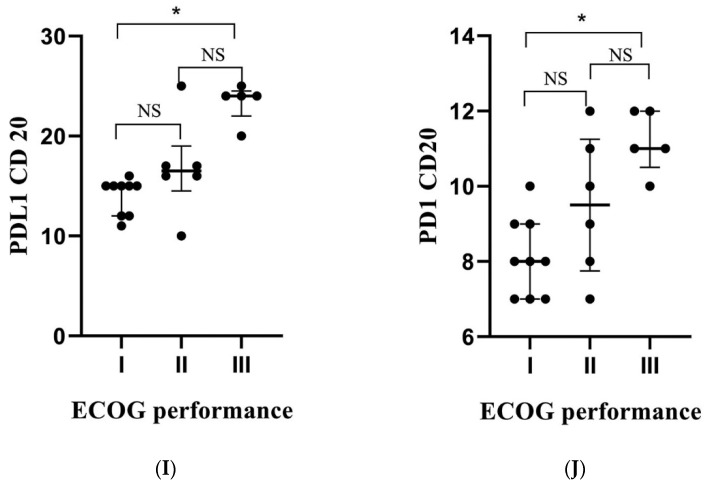
Percentage of PD-L1+CD20+ and PD-1+CD20+ in pre-therapy DLBCL patients with B symptoms (**A**,**B**); bone marrow involvement (**C**,**D**); DLBCL type (**E**,**F**); Ann Arbor stage (**G**,**H**) and ECOG performance (**I**,**J**). Each dot represented one patient. In total, 20 DLBCL patients were involved. NS: not significant; GCB: germinal center B-cell; ECOG: Eastern Cooperative Oncology Group; -ve: negative; +ve: positive. *p*-value is shown. * *p* < 0.05.

PD-L1+CD20+ % and PD-1+CD20+ % were analyzed in pre-therapy DLBCL patients with or without clinicopathological criteria. As for B symptoms and bone marrow involvement, PD-L1+CD20+ % and PD-1+CD20+ CD20+ % had a significant increase in DLBCL patients with B symptoms and bone marrow involvement (*p* < 0.001, *p* = 0.001, *p* < 0.001 and *p* = 0.001, Figure 3A–D). Similarly, patients with non-GCB subtypes presented higher PD-L1+CD20+ % and PD-1+CD20+ % than those with GCB subtypes (*p* < 0.001 and *p* = 0.009, Figure 3E,F).

Percentages of PD-L1+CD20+ in pre-therapy patients with different stages were investigated. Data revealed that DLBCL patients with stages I and II did not have significant changes in the percentages of cells, whereas DLBCL patients with stages III and IV presented higher PD-L1+CD20+ %, in which stage IV revealed significantly increased percentages of PD-L1+CD20+ cells than stages I and II (*p* = 0.001) (Figure 3G). DLBCL patients with stage IV also presented significantly higher percentages of PD-1+CD20+ cells than those with primary stages (*p* = 0.003, Figure 3H). Furthermore, patients with poor ECOG performance presented significantly higher PD-L1+CD20+ % and PD-1+CD20+ % than those with satisfactory performance (*p* = 0.003 and *p* = 0.009, Figure 3I,J). No significant correlation was observed between age or extranodal involvement and these proteins’ expression (Supplementary Table S1).

**Figure 4:**
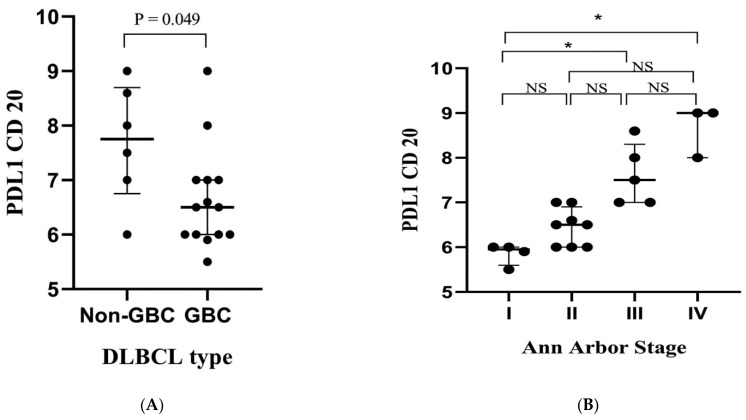
Percentage of PD-L1+CD20+ in post-therapy DLBCL patients. DLBCL type (**A**); Ann Arbor stage (**B**). Each dot represented one patient. In total, 20 DLBCL patients were involved. GCB: germinal center B-cell; NS: not significant. *p*-value is shown. * *p* < 0.05.

Additionally, the clinical relevance of these genes in post-therapy DLBCL patients was investigated. The results showed that patients with the GCB type presented lower percentages of PD-L1+CD20+ cells than those with the non-GCB subtype (*p* = 0.045, Figure 4A). Similarly, PD-L1+CD20+ % cells were significantly lower in stage I than stages III and IV (*p* = 0.001, Figure 4B). No significant associations were detected in post-therapy patients regarding other clinicopathological criteria. Furthermore, there was no significant correlation between percentages of PD-1+CD20+ and these criteria (Supplementary Table S2).

### 3.5. The Predictive and Diagnostic Efficacies of Circulating PD-L1+CD20+ and PD-1+CD20+ Cells

**Figure 5:**
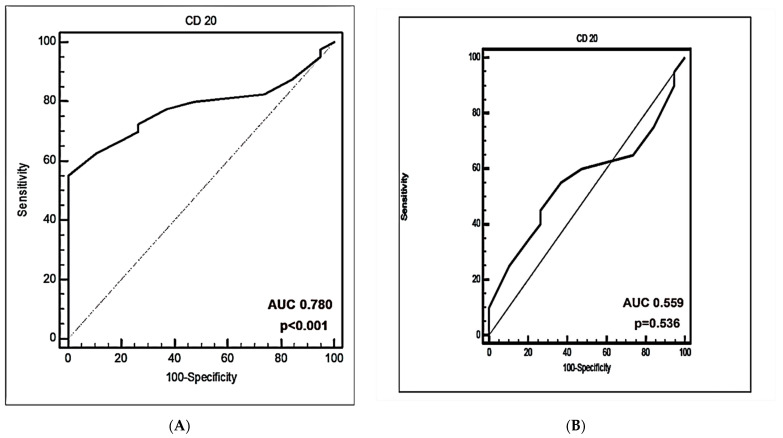

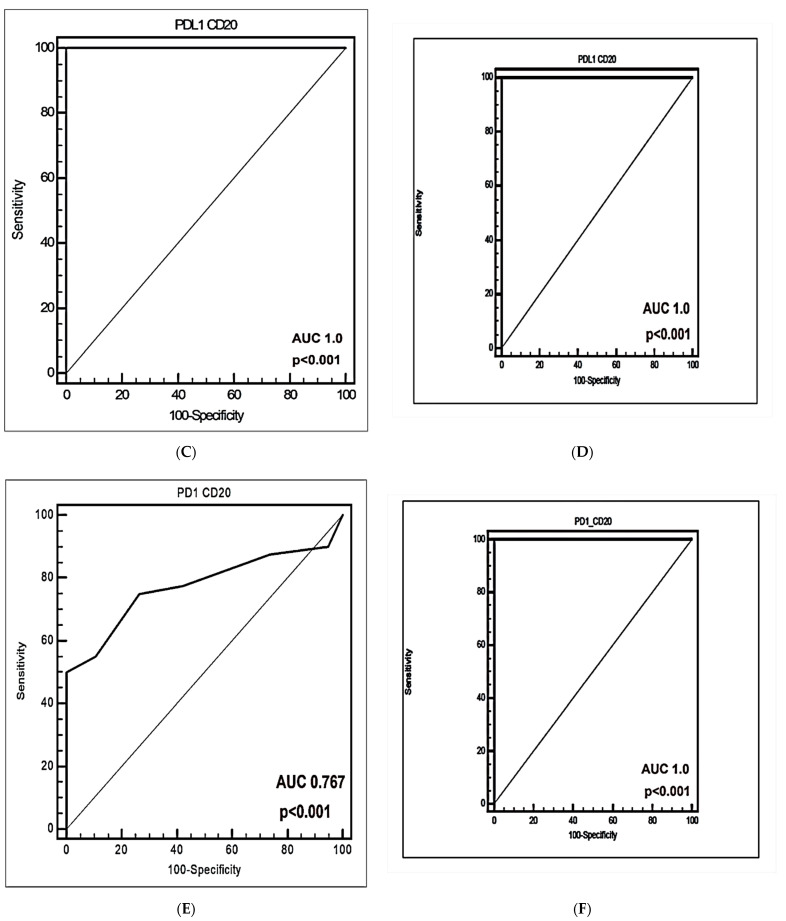
Receiver operating characteristic (ROC) curve performance for prediction and diagnosis of DLBCL. (**A**,**B**) ROC curves of percentages of CD20+ cells. (**C**,**D**) ROC curves of percentages of PD-L1+CD20+ cells. (**E**,**F**) ROC curves of percentages of PD-1+CD20+ cells (%).

The receiver operating curve (ROC) analysis was used to detect the cut-offs with the best predictive value. ROC curves of CD20+ %, PD-L1+CD20+ %, and PD-1+CD20+ % for detecting patients with DLBCL from healthy controls were illustrated in Figure 5.

The area under the curve (AUC) of CD20+ % was 0.780 for predicting patients with DLBCL from patients with normal controls [*p* < 0.001] (Figure 5A). Additionally, AUC of PD-L1+CD20+ % was 1.0 for predicting patients with DLBCL from healthy controls [*p* < 0.001], however, PD-1+CD20+ % AUC was 0.767 for identifying patients with DLBCL from healthy volunteers (*p* < 0.001) (Figure 5C,E). The sensitivity and specificity of PD-L1+CD20+ % was 100 %, while was 75% and 73.68% for PD-1+CD20+ %. The cut-off values of CD20+ %, PD-L1+CD20+ % and PD-1+CD20+ % were ≤22, >1.1 and >0.8, respectively.

In newly diagnosed patients, the specificity and sensitivity of PD-L1+CD20+ % and PD-1+CD20+ % was 100%, while was 55% and 63.16%, respectively, for CD20+ %. The discriminative cutoff of CD20+ % was ≤22, with AUC 0.559 (Figure 5B). The cutoff of PD-L1+CD20+ % was >1.1, with an AUC value of 1.0 (*p* < 0.001) (Figure 5D). The AUC value of PD-1+CD20+ % was 1.0 with cut-off > 1.2 (*p* < 0.001) (Figure 5F).

## 4. Discussion

DLBCL is an aggressive NHL with a standard treatment that depends on CHOP-like cytotoxic chemotherapy. The CHOP therapy has improved DLBCL prognosis, where about 65% of DLBCL patients entered into complete remission; however, 30–40% of patients developed relapsed/refractory (R/R) disease [29]. Recently, many therapies that have emerged in DLBCL include PD-L1/PD-1 inhibitors, but with a limited response [55]. It was shown that CD20+ cells might have a role in resistance to chemotherapy [26]. There remains no data regarding the PDL-1 and PD-1 expression in peripheral CD20 cells in DLBCL. Heterogeneity of PD-L1/PD-1 expression in DLBCL tumor cells needs to identify new markers for the prediction and diagnosis of DLBCL [56]. No previous studies were focused on analyzing PD-L1+CD20+ % and PD-1+CD20+ % within DLBCL.

This study identified PD-L1/PD-1 expression in peripheral CD20+ cells in DLBCL using flow cytometric analysis. Many studies had used the IHC method, but PD-L1 expression was not consistent owing to these assays being dependent on cutoff values, the specific type of antibodies, companies, and companion instruments used [38,57,58]. This study revealed that PDL-1/PD-1 expression in circulating CD20+ cells was highly significantly different from that in healthy volunteers. PD-L1+CD20+ % and PD-1+CD20+ % were greatly increased in newly diagnosed ones. Previously, it was suggested that PD-1+ B lymphocytes might reduce T cells expansion and that PD-L1 had a suppressive role orchestrated by PD-1+ B lymphocytes [59]. Xiao et al. [60] detected PD-1-expressing B lymphocytes with tumorigenic activity. Similar to this study, peripheral PD-1+CD20+ were rare in healthy controls [59].

Previously, PD-L1-expressing B cells were reported as a mechanism for humoral immunity suppression [61]. Previous reports showed that PD-L1 might inhibit the activation of B cells [62]. Brahmer et al. had suggested that PD-L1 antibodies therapy might relieve the PD-L1 inhibitory effect on B cells. Previous studies found that B lymphocytes suppress anti-tumor immune response [63].

After six cycles of CHOP therapy, CD20 %, PD-L1+CD20+ %, and PD-1+CD20+ % were significantly lower in post-therapy patients than pre-therapy ones, suggesting that peripheral CD20 may have a role in detecting post-therapy DLBCL patients. The reason for this observation may be the reduction in the lymph node tumor and radiotherapy treatment. Eradicating PD-L1+ CD20+ or PD-1+ CD20 cells as therapy should be examined in DLBCL. Previous reports revealed that the expression level of PDL-1 positive rate and PD-1+ B cells was reduced significantly in complete remission [61,64]. The data revealed that peripheral CD20+ cells play a significant role in DLBCL pathogenesis and highlights the significance of lowering PD-L1+CD20+ % and PD-1+CD20+ % to overcome DLBCL. 

Interestingly, the results revealed a statistically significant increase in PD-L1+CD20+ % in post-therapy patients in comparison to healthy volunteers. Herbst et al. [65] had reported no significant correlation between efficacy of therapy and PD-L1 expression. However, post-therapy patients had a statistically insignificant difference between PD-1 expression in peripheral CD20 cells and the healthy control group, which might be explained by the fact that patients could be benefitting from CHOP therapy in decreasing PD-1+ B+ percentages. Moreover, radiotherapy and tumor removal could help with the eradication of PD-1+CD20+ cells. This study involved DLBCL patients treated with CHOP chemotherapy which might affect CD20 cells and PD-1 but not PD-L1 expression in peripheral CD20 cells. 

Upon correlating PDL-1 expression with laboratory quantitative variables, CD20+ and PDL-1+CD20+ showed a significant negative correlation with serum albumin in newly diagnosed DLBCL patients. These findings suggest that there are close relationships between PDL-1/PD-1 expression levels and the prognosis and severity of the disease. Additionally, CD20+ cells and PD-L1+CD20 cells are associated with serum total bilirubin. It was suggested that increased or altered expression of PDL-1 could cause self-tolerance disruption leading to autoimmunity, especially autoimmune liver disease [66]. These results supported that PD-L1 and CD20 expression might be important for DLBCL patients’ identification with a higher risk of lymphoma progression.

The study revealed a highly significant positive association between PD-L1+CD20+ % and PD-1+CD20+ % in DLBCL. The study suggested that both cells might be engaged in DLBCL pathogenesis. The mechanism is unknown, but it might need further study in the future.

The reported associations in this study supported the significant correlations between high PD-L1+CD20+ %, PD-1+CD20 %, and bad prognosis factors such as elevated LDH levels. These results were consistent with other studies [67]. This positive association suggested the importance of PD-1+CD20+ % as a potential biomarker in DLBCL patients.

The data assumed highly significant correlations between high percentages of PD-1+CD20+ cells and splenomegaly. Extramedullary involvement and HSM may be signs of dissemination or severity of the disease, underscoring the importance of this mechanism for cancer progression and metastasis. These results supported that PD-1 expression in CD20 cells might be important for DLBCL patient identification with a high risk of lymphoma progression. In another study, expression of PD-1 was suggested to associate with a bad prognosis in DLBCL [68].

The study revealed that PD-L1+CD20+ % and PD-1+CD20+ % were significantly increased in patients with advanced disease stages (Figure 3), assuming that these cells might act as indicators of DLBCL progression. Moreover, no significant changes in percentages of cells between stage I and stage II were observed, assuming that PD-L1+CD20+ % and PD-1+CD20+ % may not have major roles at primary DLBCL stages. Previous reports have identified that PD-L1/PD-1 might be associated with DLBCL progression [69,70]. The study investigated PD-L1+CD20+ % and PD-1+CD20+ % with bone marrow involvement and B symptoms and identified that expression of PD-L1/PD-1 in CD20+ cells was increased in DLBCL patients with these clinical symptoms (Figure 3). Previous reports revealed that B symptoms and bone marrow involvement indicate a poor prognosis of DLBCL [71]. The data indicate that PD-L1+CD20+ and PD-1+CD20+ cells might be involved in DLBCL prognosis. However, the mechanism of how these cells may affect the progression and the development of DLBCL remains unclear. Further studies are required.

PD-L1+CD20+ % and PD-1+CD20+ % were higher in the non-GCB type of DLBCL, compared with GCB-type, which had low percentages of PD-L1+CD20+ and PD-1+CD20+ cells. Previous studies have mentioned that the non-GCB DLBCL type had higher PD-L1 expression than the GCB DLBCL type [72,73,74]. In contrast, there were no significant differences regarding age and extranodal involvement in newly diagnosed DLBCL patients.

In this study, decreased PD-L1+CD20+ % was associated significantly with the primary disease stages and GCB-DLBCL in post-therapy DLBCL patients. However, this was not statically significant for PD-1 expression of CD20 cells. This suggests that PD-L1+CD20+ cells can be used as a biomarker for disease severity and prognosis.

No previous studies used ROC curves to discuss the significance of PD-L1+CD20+ % and PD-1+CD20+ % in distinguishing between healthy controls and patients with DLBCL and discriminating newly diagnosed patients from healthy volunteers. In this study, the performance of the markers in differentiating DLBCL from normal controls was evaluated. PD-L1+CD20+ % and PD-1+CD20+ % improved the prediction of DLBCL patients. PD-L1+CD20+ and PD-1+CD20+ cut-off values of >1.1 and >1.2 were successful in predicting DLBCL. The use of PD-L1+CD20+ % improved the predictive performance (AUC = 1 versus 0.780 in CD20+ and 0.776 in PD-L1+CD20), with a sensitivity and specificity of 100% versus (77.50 and 63.16%) for CD20+ % and (75 and 73.68%) for PD-1+CD20+ %. The use of PD-L1+CD20+ percentages outperformed CD20+ and PD-1+CD20+ in discriminating DLBCL from normal controls. These data are vital as the PD-L1 cutoff applied for DLBCL (ranges from 5 to about 39%) and this cutoff differs greatly [38,40]. Therefore, having an improved diagnostic marker for DLBCL needs a critical priority.

The AUC value of PD-L1+CD20+ for detecting newly diagnosed DLBCL from normal controls was also as high as 1.0 and was similar to PD-1+CD20+ with a cutoff value for PD-L1+CD20+ >1.1 and for of PD-1+CD20+ >1.2. The sensitivity and specificity of PD-L1+CD20+ for differentiating pre-therapy DLBCL from healthy volunteers were 100% too. These findings reported that the diagnostic performance of PD-L1+CD20+ was superior to that of PD-1+CD20+ in diagnosing DLBCL.

According to this study, measuring the percentages of PD-L1+CD20+ cells might be a valuable diagnostic tool for the prediction and diagnosis of DLBCL. Based on these data, the study proposed that the use of flow cytometry for assessing PDL-1/PD-1 positive peripheral CD20 cells could help with the identification of DLBCL. 

## 5. Study Limitations

PD-L1+CD20+ % and PD-1+CD20 % cells had not been assessed in tumor cells. However, Kleinovink et al. [75] had revealed that expression of PD-L1 in tumor cells could not identify a positive PD-1/PD-L1 inhibitors outcome. Moreover, future studies will follow up with the patients and use a variety of methods to assess the CD20 cell function in DLBCL patients. Follow-up for DLBCL patients was not performed. The correlations between PD-1/PD-L1 expression in CD20 cells and disease recurrence or overall survival were not assessed. Future studies will follow up with the patients to investigate these correlations.

## 6. Conclusions

The results revealed that circulating PD-L1+CD20+ and PD-1+CD20+ cells may represent a companion biomarker for DLBCL and may reflect the severity of DLBCL. Strategies targeting CD20 cells expressing PD-L1 or/and PD-1 may increase the efficacy of PD-L1/PD-1 blockade immunotherapy in DLBCL. Circulating PD-L1+CD20+ cells are highly specific and sensitive for DLBCL diagnosis.

## Figures and Tables

**Table 1:** The comparison between normal controls and DLBCL patients regarding demographic and clinical data.

KERRYPNX		Control	DLBCL	*p*-Value
		*N* = 19	*N* = 40	
Age	Median	45	46.5	0.955
	IQR	(30–57)	(34–55.8)	
Sex	Male	9 (47.4%)	20 (50%)	0.850
	Female	10 (52.6%)	20 (50%)	
Hepatomegaly	No	19 (100%)	20 (50%)	<0.001 **
	Yes	0 (0%)	20 (50%)	
Splenomegaly	No	19 (100%)	24 (60%)	<0.001 **
	Yes	0 (0%)	16 (40%)	
Treatment	None		20 (50%)	
	CHOP		20 (50%)	

*N*: Number; IQR: Interquartile range. A Mann-Whitney test was used for quantitative data. The Chi-square test and Fisher exact test were used for qualitative data. High significant differences are identified with asterisks (**) (*p* < 0.001).

**Table 2:** Comparison between normal controls and patients regarding CD20+ %, PD-L1+CD20+ %, and PD-1+CD20+ %.

		Control	DLBCL	*p*-Value
		*N* = 19	*N* = 40	
CD20+ %	Median	24	14.5	0.001 *
	IQR	(20–25)	(10–22)	
PDL1+CD20+ %	Median	1	9.5	<0.001 **
	IQR	(0.8–1)	(6.7–16)	
PD1+CD20+ %	Median	0.7	4.1	0.001 **
	IQR	(0.6–1)	(0.9–9)	

N: Number; IQR: Interquartile range. The *p*-values were calculated using Mann Whitney test. Significant differences are identified with asterisks (*). (*p* < 0.001). High significant differences are identified with asterisks (**) (*p* < 0.001).

**Table 3:** CD20+ %, PD-L1+CD20+ %, and PD-1+CD20+ % in DLBCL patients and healthy subjects.

		Controls (I)	Pre-Therapy DLBCL (II)	Post-Therapy DLBCL (III)	*p*		
		*N* = 19	*N* = 20	*N* = 20	I vs. II	I vs. III	II vs. III
CD20+ %	Median	24	22	10	0.525	<0.001 *	<0.001 **
	IQR	(20–25)	(18.3–26.8)	(9.6–11.8)			
PDL-1+CD20+ %	Median	1	16	6.8	<0.001 **	<0.001 *	<0.001 **
	IQR	(0.8–1)	(15–23)	(6–7.9)			
PD-1+CD20+ %	Median	0.7	9	0.9	<0.001 **	0.575	<0.001 **
	IQR	(0.6–1)	(8–11)	(0.6–1)			

Group I: Healthy volunteers; Group II: Pre-therapy DLBCL patients; Group III: Patients completed 6 CHOP cycles. N: Number; IQR: Interquartile range. Kruskal Wallis test was used for the analysis of data between the three groups followed by pairwise comparisons between every two groups using Bonferroni correction. Significant differences are identified with asterisks (*). High significant differences are identified by an asterisk (**) (*p* < 0.001).

**Table 4:** Low versus high CD20+ %, PD-L1+CD20+ % and PD-1+CD20+ %.

		Control (I)	Pre-Therapy DLBCL (II)	Post-Therapy DLBCL (III)	*p*		
		*N* = 19	*N* = 20	*N* = 20	I vs. II	I vs. III	II vs. III
CD20+ %	Low (<19)	4 (21.1%)	7 (35%)	20 (100%)	0.333	<0.001 **	<0.001 **
	High (>19)	15 (78.9%)	13 (65%)	0 (0%)			
PDL-1+CD20+ %	Low (<6.8)	19 (100%)	0 (0%)	10 (50%)	<0.001 **	<0.001 **	<0.001 **
	High (>6.8)	0 (0%)	20 (100%)	10 (50%)			
PD-1+CD20+ %	Low (<1)	17 (89.5%)	0 (0%)	18 (90%)	<0.001 **	1.0	<0.001 **
	High (>1)	2 (10.5%)	20 (100%)	2 (10%)			

Group I: Healthy volunteers; Group II: Pre-therapy DLBCL patients with no treatment; Group III: Patients completed 6 CHOP cycles. *N*: Number. Fisher exact test was used for the analysis between the groups and between every two groups. (** *p* < 0.001) identifies highly significant differences.

**Table 5:** Correlation of high CD20+ %, PD-L1+CD20+ %, PD-1+CD20+ % cells and DLBCL patient disease characteristics.

	High CD20+ %		High PD-L1+CD20+ %		High PD-1+CD20+ %	
	r	*p*-Value	r	*p*-Value	r	*p*-Value
High PD-L1+CD20+ % ^(P)^	0.401	0.010 *				
High PD-1+CD20+ % ^(P)^	0.628	<0.001 **	0.638	<0.001 **		
L D H ^(P)^ U/L	0.479	0.002 *	0.365	0.021 *	0.549	<0.001 **
Random glucose ^(P)^ (mg/dL)	0.019	0.910	0.368	0.020 *	0.152	0.348
Splenomegaly ^(S)^	0.235	0.151	0.251	0.123	0.354	0.027 *

LDH: Lactate Dehydrogenase. (* *p* ≤ 0.05) identifies significant differences). ^(P)^ Pearson’s correlation; ^(S)^ Spearman’s correlation. (** *p* < 0.001) identifies highly significant differences.

## Data Availability

The datasets generated during and/or analyzed during the current study are not publicly available but are available from the corresponding author on reasonable request.

## Supplementary Materials

The following supporting information can be downloaded at: https://www.mdpi.com/article/10.3390/antib11010015/s1, Table S1: Association of PD-L1+CD20+ % and PD-1+CD20+ % with the clinicopathological characteristics of pre-therapy DLBCL patients. Table S2: Association of PD-L1+CD20+ % and PD-1+CD20+ % with the clinicopathological characteristics of post-therapy DLBCL patients.
