# Supplementary material for: Diagnostic Performance of PD-L1 versus PD-1 Expression in Circulating CD20 Cells in Diffuse Large B-Cell Lymphoma
Source: Antibodies (Basel). 2022 Feb 16;11(1):15. doi: 10.3390/antib11010015 (PMC8884023; doi:10.3390/antib11010015)
Supplement: Supplementary file 1 [file antibodies-11-00015-s001.zip › antibodies-1448860-supplementary.pdf]

		N	PDL1+CD20+	P-value	PD1+CD20+	P-value
Ann Arbor Stage	I	4	13.5 [a] (12-15)	0.001*	7.5 [a] (7-8)	0.003*
	II	5	15 [a] (10.5-15)		8 [a] (7-9.5)	
	III	5	16 (16-17)		9 (8.5-10.5)	
	IV	6	24 [b] (23-25)		11.5 [b] (10.8-12)	
B symptoms	-Ve	8	13.5 (11.3-15)	<0.001**	7.5 (7-8)	0.001*
	+Ve	12	18.5 (16-24)		10.5 (9.3-11.8)	
Bone marrow involvement	-Ve	13	15 (12-16)	<0.001**	8 (7-9)	0.001*
	+Ve	7	24 (20-25)		11 (10-12)	
Extranodal involvement	-Ve	3	15 (15-)	0.830	8 (8-)	0.957
	+Ve	17	16 (13.5-22)		9 (7.5-11)	
DLBCL Type	Non-GCB	9	24 (16.5-24.5)	<0.001**	11 (9-12)	0.009*
	GCB	11	15 (12-15)		8 (7-10)	
Age	<60	15	16 (12-20)	0.310	9 (7-11)	0.595
	≥60	5	17 (15-24.5)		10 (8.5-10.5)	
	+Ve	12	16.5 (15-24)		10 (7.3-11)	
ECOG performance	I	9	15 [a] (12-15)	0.003*	8 [a] (7-9)	0.009*
	II	6	16.5 (14.5-9)		9.5 (7.8-11.3)	
	III	5	24 [b] (22-24.5)		11 [b] (10.5-12)	

Supplementary Table S1. Association of PD-L1+CD20+ % and PD-1+CD20+ % with the clinicopathological characteristics of newly diagnosed patients with DLBCL. GCB: germinal center B-cell; N: Number; ECOG: Eastern Cooperative Oncology Group. Mann Whitney test was used to compare the differences between the two groups. Kruskal Wallis test was used to compare the difference between more than 2 groups followed by pairwise comparisons between every two groups using Bonferroni correction. * Significant difference at p-value < 0.05. High significant differences are identified with asterisks (**) (p< 0.001). Superscripts with different small letters refer to the significant difference between every two groups at a p-value < 0.05.

		N	PDL1+CD20+	P-value	PD1+CD20+	P-value
**Ann Arbor Stage**	I	4	6 [b] (5.6-6)	0.001*	0.7 (0.7-0.9)	0.591
	II	8	6.5 (6-6.9)		0.9 (0.5-1)	
	III	5	7.5 [a] (7-8.3)		0.7 (0.5-1.1)	
	IV	3	9 [a] (8-)		1 (1-1)	
**DLBCL Type**	Non-GBC	6	7.8 (6.8-8.7)	0.049*	1 (0.5-1.1)	0.667
	GBC	14	6.5 (6-7)		0.8 (0.6-1)	
**Age**	<60	17	7 (6-7.8)	0.748	1 (0.6-1)	0.620
	≥60	3	6.6 (6-)		0.8 (0.5-)	
	+Ve	5	8 (5.8-8.8)		1 (0.6-1.1)	
**ECOG performance**	I	16	6.8 (6-8)	0.473	1 (0.7-1)	0.140
	II	4	6.5 (5.9-7.4)		0.6 (0.5-0.9)	

Supplementary Table S2. Association of PD-L1+CD20+ % and PD-1+CD20+ % with the clinicopathological characteristics of post-therapy DLBCL patients**.** N: Number; ECOG: Eastern Cooperative Oncology Group; GCB: germinal center B-cell. Mann Whitney test was used to compare the differences between the two groups. Kruskal Wallis test was used to compare the difference between more than 2 groups followed by pairwise comparisons between every two groups using Bonferroni correction. * Significant difference at p-value < 0.05. High significant differences are identified with asterisks (**) (p< 0.001). Superscripts with different small letters refer to the significant difference between every two groups at a p-value < 0.05.
